# Supplementary material for: Spectral tuning and deactivation kinetics of marine mammal melanopsins
Source: PLoS One. 2021 Oct 15;16(10):e0257436. doi: 10.1371/journal.pone.0257436 (PMC8519484; doi:10.1371/journal.pone.0257436)
Supplement: S1 Table — Forward and reverse primers for PCR amplification of full-length bowhead whale Opn4 and partial coding domain for other cetacean Opn4 sequences including North Atlantic right whale, bottlenose dolphin and harbor porpoise. (DOCX) [file pone.0257436.s001.docx]

| **Mammal** | **Forward** | **Reverse** |
| --- | --- | --- |
| **West Indian manatee** | 5’-CCA ACT GCA AGA ATT CAT GAA CCC TTG GGG GCC-3’ | 5’-GGA ATT TGC GGC CGC TTA GGC AGG CGC CAC TTG-3’ |
| **Bowhead whale** | 5’-CCG GAA TTC ATG AAC TCA CCT TTG GGG ACC GGA-3’ | 5’-TTT TCC TTT TGC GGC CGC TTA AGC TGG TGC AAC-3’ |
| **Other cetaceans** | 5’-CAG GGC TCA GGC TGC TGC CT-3’  5’- GTG GTG TCA GTA ACT CAG CA -3’ | 5’-CAC CTC RCT CTC NGA GCC CAG G-3’  5’- TCC CTG GGC TCT GAG AGC GA-3’ |

**S1 Table. Oligonucleotide primers for PCR.** Forward and reverse primers for PCR amplification of full-length bowhead whale Opn4 and partial coding domain for other cetacean Opn4 sequences including North Atlantic right whale, bottlenose dolphin and harbor porpoise.
