# Supplementary material for: Spectral tuning and deactivation kinetics of marine mammal melanopsins
Source: PLoS One. 2021 Oct 15;16(10):e0257436. doi: 10.1371/journal.pone.0257436 (PMC8519484; doi:10.1371/journal.pone.0257436)
Supplement: S2 Table — Temperatures for denaturation, annealing and extension for the amplification of Opn4 coding sequences from bottlenose dolphin, harbor porpoise and North Atlantic right whale retinal cDNA. Reactions contained forward and reverse primers (see above) and were carried out in a 25 μl mixture containing 1 μM of each primer, 2 μl of reverse transcribed single-stranded cDNA, and Amplitaq Gold master mix (Invitrogen, Carlsbad, CA). Annealing Tm were from NCBI Primer-BLAST for each primer pair. (DOCX) [file pone.0257436.s002.docx]

| **Cycle Temperatures** | | **Cycle Settings** |  |
| --- | --- | --- | --- |
|  |  |  |  |
| 94 ºC |  | 5 min |  |
| 94 ºC |  | 1 min | --- 30 cycles |
| 55-63 ºC |  | 30 sec |  |
| 72 ºC |  | 1 min |  |
| 4 ºC |  | Hold |  |

**S2 Table.** **PCR cycling parameters.** Temperatures for denaturation, annealing and extension for the amplification of Opn4 coding sequences from bottlenose dolphin, harbor porpoise and North Atlantic right whale retinal cDNA. Reactions contained forward and reverse primers (see above) and were carried out in a 25 µl mixture containing 1 µM of each primer, 2 μl of reverse transcribed single-stranded cDNA, and Amplitaq Gold master mix (Invitrogen, Carlsbad, CA). Annealing T_m_ were from NCBI Primer-BLAST for each primer pair.
