# Supplementary material for: Spectral tuning and deactivation kinetics of marine mammal melanopsins
Source: PLoS One. 2021 Oct 15;16(10):e0257436. doi: 10.1371/journal.pone.0257436 (PMC8519484; doi:10.1371/journal.pone.0257436)
Supplement: S3 Table — Temperatures for denaturation, annealing and extension for the amplification of West Indian manatee and bowhead whale opn4 coding sequences. (DOCX) [file pone.0257436.s003.docx]

| **Cycle Temperatures** | | **Cycle Settings** |  |
| --- | --- | --- | --- |
| **Indian manatee** | **Bowhead whale** |  |  |
| 95 ºC | 95 ºC | 2 min |  |
| 95 ºC | 95 ºC | 15 sec | --- 30 cycles |
| 62 ºC | 61.8 ºC | 30 sec |  |
| 68 ºC | 68 ºC | 1 min 40 sec |  |
| 4 ºC | 4 ºC | Hold |  |

**S3. Table. PCR cycling parameters.** Temperatures for denaturation, annealing and extension for the amplification of West Indian manatee and bowhead whale opn4 coding sequences.
