# Supplementary material for: Spectral tuning and deactivation kinetics of marine mammal melanopsins
Source: PLoS One. 2021 Oct 15;16(10):e0257436. doi: 10.1371/journal.pone.0257436 (PMC8519484; doi:10.1371/journal.pone.0257436)
Supplement: S4 Table — GenBank accession numbers used to generate the tree shown in Fig 2 and in commented on in the Discussion. (DOCX) [file pone.0257436.s004.docx]

| **Opsin** | **Common name** | **Scientific name** | **Accession number** |
| --- | --- | --- | --- |
| **Rod opsin (Rh1):** | | | |
|  | zebrafish | *Danio rerio* | NM_131084 |
|  | mouse | *Mus musculus* | NM_145383 |
|  | human | *Homo sapiens* | NM_000539 |
|  | Nile tilapia | *Oreochromis niloticus* | AY775108 |
|  | Arctic lamprey | *Lethenteron japonicum* | M63632 |
|  |  |  |  |
| **Rod opsin-like 2 (Rh2):** | | | |
|  | zebrafish |  | NM131253 (Rh2.1) |
|  |  |  | NM182891 (Rh2.2) |
|  |  |  | NM182892 (Rh2.3) |
|  |  |  | NM131254 (Rh2.4) |
|  | Nile tilapia |  | AF247124 |
|  |  |  |  |
| **Short-wavelength sensitive-1 (SWS1):** | | | |
|  | human |  | NM_001708 |
|  | mouse |  | NM_007538 |
|  | zebrafish |  | NM_131319 |
|  | Nile tilapia |  | XM_003448756 |
|  | pouched lamprey | *Geotria australis* | AY366495 |
|  |  |  |  |
| **Short-wavelength sensitive-2 (SWS2):** | | | |
|  | zebrafish |  | NM_131192 |
|  | Nile tilapia |  | AF247116 (SWS2A) |
|  |  |  | AF247120 (SWS2B) |
| **Long-wavelength sensitive (LWS):** | | | |
|  | Nile tilapia |  | XM_003442629 |
|  | zebrafish |  | KT008400 (LWS1)  KT008401 (LWS2) |
|  | mouse |  | AF190672 |
|  | human |  | NM_000513 (LWS-MW) |
|  |  |  | NM_020061 (LWS-LW) |
|  | pouched lamprey |  | AY366491 |
|  |  |  |  |
| **Retinal pigment epithelium-derived rhodopsin (RRh):** | | | |
|  | human |  | NM_006583 |
|  | mouse |  | NM_009102 |
|  | Nile tilapia |  | XM_003458164 |
|  | zebrafish |  | NM_001004654 |
|  |  |  |  |
| **Melanopsin (Opn4):** | | | |
|  | elephant shark | *Callorhinchus milii* | NM_001292428 (Opn4m1) |
|  |  |  | NM_001292116 (Opn4m2) |
|  |  |  | NM_001292471(Opn4x-long) |
|  | whale shark | *Rhincodon typus* | XM_020523467 |
|  | mouse |  | NM_013887 |
|  | human |  | NM_033282 |
|  | salmon | *Salmo salar* | XM_014153974 |
|  | chicken | *Gallus gallus* | NM_001044653 |
|  | frog | *Xenopus laevis* | XM_018254470 |
|  | alligator | *Alligator mississippiensis* | XM_019488093 |
|  | small-eared galago  thirteen-lined ground squirrel | *Otolemur garnettii*  *Ictidomys tridecemlineatus* | XM_003803012  XM_021720398 |

**S4. Table. Accession numbers.** GenBank accession numbers used to generate the tree shown in Figure 2 and in Discussion.
