# Supplementary material for: Spectral tuning and deactivation kinetics of marine mammal melanopsins
Source: PLoS One. 2021 Oct 15;16(10):e0257436. doi: 10.1371/journal.pone.0257436 (PMC8519484; doi:10.1371/journal.pone.0257436)
Supplement: Attachment — Submitted filename: Response to Reviewers.pdf [file pone.0257436.s006.pdf]

August 27, 2021

Dear Dr. Neuhauss,

We the authors have revised our manuscript (Manuscript ID PONE-D-21-18150) entitled, ***Spectral Tuning and Deactivation Kinetics of Marine Mammal Melanopsins*** for review by you as well as by the referees who enthusiastically agreed to review the revised manuscript. We have revised the manuscript by addressing the comments and concerns of the Academic Editor, and referees #1 and #2. The comments and concerns of the Academic Editor and the referees were extremely helpful in crafting a more sophisticated and concise manuscript and we thank the Academic Editor and referees for their time and well-thought-out critiques. Please note that all line numbers (denoted L) mentioned in this rebuttal refer to the line numbers from the original manuscript (file name: Manuscript) as do the reference numbers.

Comments and Responses

<u>Academic Editor</u>

Comment 1. Data available upon request. When filling out the submission for review, this option was a little confusing. Our data (DNA sequences) will be available and uploaded to NCBI when the manuscript is accepted for publication. Sequence accession numbers will be provided when available.

<u>Reviewer #1</u>

Comment 1. L133. Refs? The sentence ending on L133 proposes two hypotheses pertaining to mechanisms that may counter photoreceptor bleaching in bright light conditions. To our knowledge, there has been no research on the selection for rod visual pigments or mutants that reconstitute chromophore significantly faster. Our current work, of course, pertains to the second hypothesis proposed on L133, a compensatory mechanism to maintain an extended PLR wherein the pupil dilates considerably slower.

Comment 2. L144. The authors do not explicitly mention the phylogenic proximity of some species with the cow used as a reference. It may not be obvious to the readership. We agree with Reviewer #1's comment as this phylogenic proximity was buried at the end of Figure legend 2. Even-toed ungulates, like cow, and cetaceans belong to the same order, Cetartiodactyla. We have edited the Materials and Methods as follows: "Domestic cow *Opn4* (*Bos Taurus*, GenBank accession no. NM_001192399) was used as a query sequence in nucleotide blast analyses to identify orthologs from other Cetartiodactyla marine mammal genomes including…"

Comment 3. L223. What was the irradiance of the flashes? Irradiance values from the Xenon flash lamp were not measured nor listed in the specifications of the instrument. However, a 5W Xenon flash lamp typically has irradiance values between 5-15 $mWcm^2$.

Comment 4. L373. Spectral measurements were performed "between astronomical twilight (06:35 EDT) and sunrise (07:00 EDT)". What was the reasoning behind performing the

spectral measurements only at dawn? Also, the readership would benefit from some descriptions of the marine species habitat/habits. For example: What is the amount and schedule of light sampling at the surface in the marine mammals considered? That, given the vast diversity of species considered, may be quite different. What is the underwater spectrum at the depth where these species are living? With regards to the first question (…*why sample at dawn?*), this was simply the only time available to us on that day. At the time, JIF was studying photoentrainment pertaining to circadian rhythms. In retrospect, we now would have preferred to have sampled at high noon as well as at dusk to have a full account of the solar spectral measurements. With regards to the second question (*What is the amount and schedule of light sampling at the surface in the marine mammals considered?*), this may be found in L533-555 where we state, "However, the dominant wavelengths of solar spectral irradiance present in surface water environments is around 510 nm [77, 86]", with radiance values near 1 Lw (found in reference 86). With regards to the third comment (*What is the underwater spectrum at the depth where these species are living?*), we address this along with Reviewer #2's comment ("…*some information on the spectral composition of their light environment and the illumination levels*"). Readers should be able to use the references found in lines 92-102 with regards to visual foraging ecology of marine mammals: "...marine mammal visual pigments are spectrally tuned to overlap the underwater spectral radiance associated with foraging depth [9, 12, 42-51]. Modulating the spectral sensitivity results in relatively large, blue-shifted absorbance spectra from visual pigments of deep-diving pelagic marine mammals, and relatively slightly red-shifted absorbance spectra from the visual pigments of near-coastal and riverine species, when compared to their terrestrial counterparts. Unlike terrestrial mammals, most marine mammal species reside in two spectrally distinct photic environments: the surface where they breathe and utilize broadband light spectra, and at foraging depth where they can utilize narrowband light spectra. The spectral irradiance at foraging depth is typically blue-shifted from the 500 nm region of the visible light spectrum where terrestrial Rh1 pigments maximally absorb [7, 9, 44, 45, 57-60]*"*, with reference 58 providing radiance values as a function of depth for the North Atlantic right whale. Outside of this, describing the underwater spectral qualities of light for each species is beyond the scope of this paper.

Comment 5. L389. (Ka/Ks or dN/dS ratio, also referred to as ω), not clear. We agree with Reviewer #1's comment and have edited this sentence as follows: "To investigate patterns of selection in marine mammal melanopsins, we used codon-aligned models to estimate the ratio of non-synonymous (amino-acid altering; Ka or dN) to synonymous (silent; Ks or dS) substitutions (Ka/Ks or dN/dS ratio, also referred to as ω)…"

Comment 6. L455. The authors may also want to cite Mure et al., 2016 regarding the impact of Opn4 C-terminus tail phosphorylation on the PLR. We agree with Reviewer #1 and have added this reference.

Comment 7. L455. Somasundaram et al., 2017 is referenced twice (69 and 83). This has been corrected.

Comment 8. L525. "why have the authors made no attempt of spectral measurements with the calcium assay they used to compare deactivation kinetics? The authors agree and this is

an experiment that will be performed in the future. It requires a modification of the instrument such that single monochromatic light is flashed to a select number of samples.

Comment 9. Regarding absorption peak, the authors could discuss fish melanopsins as fishes share the same habitat but do not surface. Are some fishes' melanopsin spectra known (and shifted?)? We agree with Reviewer #2 that this would be an interesting comparison and we hope to begin similar studies with marine fish. An article that interested us examined melatonin inhibition in damselfish in the presence of blue light as opposed to green or red light (Takeuchi et al. 2014. Gen. Comp. Endocrin. 204: 158-165) but did not examine the damselfish melanopsin gene or its expression. To our knowledge, the spectral tuning properties in association with calcium imaging assays of marine fish melanopsins have not been done.

Reviewer #2

Comment 1. The connection to the pupillary light response needs more supportive references. It is not entirely clear to us what Reviewer #2 is referring to with regards to "connection"? We can only assume that Reviewer #2 is referring to the "connection" between light, melanopsin expressing ipRGCs, as well as the innervation of rod and cone photoreceptors on these ipRGCs. The following 5 references directly examine the pupillary light response with regards to Opn4 expressing ipRGCs, while several other references in the manuscript describe the roles of rod and cone photoreceptors. We believe that we have included the appropriate references in the manuscript. We included a new reference per the request of Reviewer #1 (Mure et al., 2016).

Markwell EL, Feigl B, Zele AJ. Intrinsically photosensitive melanopsin retinal ganglion cell contributions to the pupillary light reflex and circadian rhythm. Clinic Exp Optomet. 2010;93(3):137-49. https://doi.org/10.1111/j.1444-0938.2010.00479.x

Keenan WT, Rupp AC, Ross RA, Somasundaram P, Hiriyanna S, Wu Z, et al. A visual circuit uses complementary mechanisms to support transient and sustained pupil constriction. eLife 2016;5: e15392 doi: 10.7554/eLife.15392

Lee SK, Sonoda T, Schmidt TM. M1 Intrinsically Photosensitive Retinal Ganglion Cells Integrate Rod and Melanopsin Inputs to Signal in Low Light. Cell Reports. 2019;29(11):3349-55. e2. https://doi.org/10.1016/j.celrep.2019.11.024

Mure LS, Hatori M, Zhu Q, Demas J, Kim IM, Nayak SK, et al. Melanopsin-encoded response properties of intrinsically photosensitive retinal ganglion cells. Neuron. 2016; 90: 1016–27. http://dx.doi.org/10.1016/j.neuron.2016.04.016

Somasundaram P, Wyrick GR, Fernandez DC, Ghahari A, Pinhal CM, Richardson MS, et al. C-terminal phosphorylation regulates the kinetics of a subset of melanopsin-mediated behaviors in mice. PNAS USA. 2017:201611893. https://doi.org/10.1073/pnas.1611893114

Comment 2. In general, the authors should comment more on the visual ecology of these species. This includes some information on the spectral composition of their light environment and the illumination levels. See Reviewer #1, Comment 4.

Comment 3. The discussion would be even more interesting by a comparison of other marine life, e.g., what is known about fish melanopsins, are there difference between surface and deep-sea fish … But this is of course at the discretion of the authors. See Reviewer #1, Comment 9.

Thank you for your consideration and please contact me for any further comments and corrections,

Sincerely,

Jeffry I Fasick, PhD
